# Discovery of Zidovudine as a cardiomyocyte protectant for doxorubicin-induced toxicity through high-throughput phenotypic drug screening

**DOI:** 10.1016/j.fmre.2023.10.010

**Published:** 2023-11-19

**Authors:** He Xu, Hao You, Jixing Gong, Ying Zhang, Jianyong Du, Xinyu Wang, Shanshan Gu, Nan Cao, Jia Wang

**Affiliations:** a Advanced Medical Technology Center, The First Affiliated Hospital, Zhongshan School of Medicine, Sun Yat-Sen University, Guangzhou 510080, China; b Center of Translational Medicine, The First Affiliated Hospital, Zhongshan School of Medicine, Sun Yat-Sen University, Guangzhou 510080, China; c Key Laboratory for Stem Cells and Tissue Engineering (Sun Yat-Sen University), Ministry of Education, Guangzhou 510080, China; d Research Center for Cardiopulmonary Rehabilitation, University of Health and Rehabilitation Sciences Qingdao Hospital (Qingdao Municipal Hospital), School of Health and Life Sciences, University of Health and Rehabilitation Sciences, Qingdao 266071, China; e Department of Immunology and Microbiology, Zhongshan School of Medicine, Sun Yat-Sen University, Guangzhou, 510080, China

**Keywords:** Doxorubicin-induced cardiotoxicity, Drug screen, FDA-approved drugs, Zidovudine, Engineering heart tissue

## Abstract

Doxorubicin (DOX) constitutes a cornerstone in cancer chemotherapy, yet its administration is associated with dose-dependent toxicity to the heart, known as doxorubicin-induced cardiotoxicity (DOX-IC). Presently, dexrazoxane stands as the sole approved drug for mitigating DOX-IC; however, its clinical application is restricted due to concerns over severe adversary effects. It is therefore urgent to discover alternative drug candidates to ameliorate DOX-IC. Here, we report the discovery of Zidovudine (ZIDO), a clinically available anti-retroviral medication, as a potent candidate to protect against DOX-IC both *in vitro* and *in vivo* by high-throughput phenotypic screening of a library of 1804 Food and Drug Administration (FDA)-approved drugs. Transcriptomic analysis reveals that ZIDO treatment significantly alleviates DOX-induced dysregulation of genes associated with cardiac function and proteotoxic stress. This study sets a paradigm towards discovering novel cardiac protective drugs through repurposing and establishes ZIDO as an agent holding promise for the treatment of DOX-IC patients.

## Graphical abstract

**Figure:**
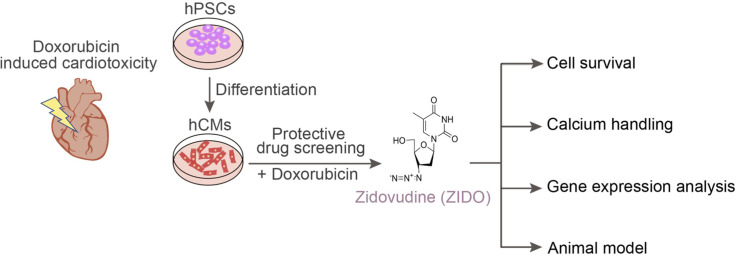

## 1 Introduction

Among the ever-growing number of cancer patients, cancer treatment associated heart disease has become the second most cause of mortality in cancer survivors [1]. Doxorubicin (DOX) ranks as one of the most widely prescribed chemotheraputic agents for a diverse array of cancer types [2], encompassing solid tumors and hematological malignancies [3]. Its clinical application dates back to the 1960s [4]. However, serious adverse impacts of DOX on the heart, spanning chronic and acute cardiotoxicity, have been documented to occur in as high as 60% of the treated adult patients [3,5,6], ultimately leading to congestive heart failure in patients receiving a cumulative dose above 550 mg/m^2^ [4,7]. Even with strict dose limitations, 14.5% of breast cancer patients have experienced cardiotoxicity when receiving the most common cumulative DOX dose of 240 mg/m^2^ [8].

Dexrazoxane (DRZ) currently stands as the sole Food and Drug Administration (FDA)-approved remedy available for mitigating doxorubicin-induced cardiotoxicity (DOX-IC) [9,10]. However, its use is restricted to adult patients with advanced or metastatic breast cancer who have received high cumulative DOX doses and necessitate continued anthracycline-based therapy to manage tumor progression [11]. Additionally, concerns regarding DRZ-associated leukemic and myelodysplastic syndrome, as well as the development of secondary malignancies significantly hamper the clinical use of DRZ [12]. It is imperative, therefore, to discover new DOX-IC protective drug candidates.

Over the past years, several studies have reported protection of DOX-IC from some candidates [[13], [14], [15], [16], [17], [18]]. However, these investigations relied on animal models that diverge significantly from humans in various functional and physiological aspects [19], and these non-human models have proven to be unsuitable for clinical translation [20]. Consequently, none of those DOX-IC ameliorating drug candidates identified through animal models have been successfully translated into clinical practice.

Recent advances in the derivation of cardiomyocytes (CMs) from human pluripotent stem cells (hPSCs) via directed differentiation provide an unprecedented opportunity to study heart diseases [21], in part due to the ease of deriving large quantities of CMs as well as bypassing the difficulties associated with obtaining tissue samples from donors. Furthermore, hPSC-derived CMs (hCMs) are also suitable for identifying and analyzing the genetic basis and molecular mechanisms of drug-induced cardiotoxicity [[22], [23], [24]]. Our laboratory is among those that have previously applied hCM-based *in vitro* model to assess drug toxicity [[25], [26], [27]]. Nevertheless, to our knowledge, there exists no report on the systematic discovery of DOX-IC protective drugs based on the human CM system.

The quest for novel drugs is a lengthy and costly endeavor [28]. We reason that systematic discovery of DOX-IC protective candidates from available drugs could represent a more favorable alternative. It is well-recognized that drug repurposing entails a reduced risk of failure and a shorter developmental timeline compared to developing a new drug [29]. Therefore, in this study, we combine the hCM platform with high-throughput drug screening technology and perform phenotypic analysis with a library of 1804 FDA-approved drugs with the aim of finding potent DOX-IC alleviating drugs. In this pursuit, we unveil Zidovudine (ZIDO), a commonly used anti-human immunodeficiency virus (HIV) medication, as a substantial attenuator of DOX-IC in both cultured human CMs and a mouse model of chronic DOX-IC, therefore providing an enticing candidate warranting further clinical development in the realm of DOX-IC management.

## 2 Materials and methods

### 2.1 Cell culture and differentiation

Human embryonic stem cell line H1 (WiCell) was maintained in E8 medium (05990, Stem Cell Technologies), with daily change of medium, on Matrigel (354277, BD Biosciences) pre-coated plates at a controlled environment of 37 °C with 5% (vol/vol) CO_2_. Cells were passaged with EDTA at approximately 70% confluency. Human cardiac differentiation was performed as previously described [25,27,30]. CMs were metabolically purified by using glucose- and sodium pyruvate-free condition (Dulbecco's modified Eagle medium (DMEM, 11966-025, Gibco) supplemented with 20 mM sodium lactate (L7022, Sigma) as previously described [27]. Subsequently, the CMs were reseeded onto Matrigel-coated plates and cultured in a specific CM culture medium comprising DMEM/F12 (C11330500BT, Gibco), 10.7 mg L^−1^ Transferrin (T0065, Sigma), 71 mg L^−1^ Vitamin C (A8960, Sigma), 14 ng mL^−1^ sodium selenite (S5261, Sigma), 38.4 mg L^−1^ insulin (91077C, Sigma), and 1 × Chemically Defined Lipid Concentrate (11905031, Gibco).

### 2.2 Isolation and culture of primary cardiomyocytes

To isolate neonatal rat cardiomyocytes (NRCMs), one-day-old Sprague-Dawley rat ventricles were minced and digested to completion with 1 mg/ml collagenase II (LS004177, Worthington) and 0.125% trypsin. NRCMs were isolated from fibroblasts through a differential seeding process. Subsequently, these isolated NRCMs were cultured in gelatin-coated tissue culture plates at a density of 2 × 10^4^ cells/cm^2^.

### 2.3 High-throughput drug screening

Purified hCMs were seeded into 384-well plates at a density of 8000 cells per well and allowed to recover in CM culture medium. Two days after seeding, the cells were subjected to treatment with 1 µM DOX (T1020, TargetMol) alone or in combination with a library of 1804 U.S FDA-approved drugs (Topscience), each at a final concentration of 5 µM. The drugs were individually added using the Tecan Freedom EVO 150 liquid Handler (Tecan). Cells were then treated for three days and subsequently stained using a Calcein-AM/PI kit (C542, DOJINDO) before being loaded onto the Operetta CLS High-Content Analysis System (PerkinElmer) for image capture. The number of live cells (Calcein-AM^+^/PI^−^) and necrotic cells (PI^+^) was quantified utilizing the Harmony 4.9 software (PerkinElmer). The “relative cell viability” was determined by the following equation:

$$\text{relative}\;\text{cell}\;\text{viability}=\frac{\;\text{live}\;\text{cell}\;\text{number}\;\text{in}\;\text{each}\;\text{treatment}\;\text{group}}{\text{live}\;\text{cell}\;\text{number}\;\text{in}\;\text{Normal}\;\text{group}}\;\times \;100\%$$

### 2.4 Apoptosis assay

Cell apoptosis was assessed with the Terminal Deoxynucleotidyl Transferase-mediated dUTP Nick End Labeling BrightRed Apoptosis Detection Kit (A113, Vazyme), adhering to the manufacturer's instructions. Cells were co-stained with the CM marker cTNT antibody (MA512960, Invitrogen) and only the cTNT^+^ CMs were included in the apoptosis analysis. Furthermore, hCMs were stained with γ-H2Ax (05-636-I, Merck) and cleaved caspase (9664, Cell signaling technology). The ratio of positive cells were calculated. Images were captured using the Operetta CLS High-Content Analysis System, and the ratio of TUNEL^+^ cells were analyzed and quantified with Harmony 4.9 software.

### 2.5 Calcium transient measurement

Human CMs were seeded onto Matrigel-coated glass coverslip and cultured overnight for recovery. Prior to measurement, the culture medium was substituted with the Tyrode's buffer, and the cells were loaded with 1 µM Fura-2 AM (F1221, Invitrogen) at 37 °C for 15 min followed by wash with the Tyrode's buffer. For the assessment of calcium transients, the cells were paced with field stimulation, employing monophasic, 10 V, 5 ms pulses, at frequencies of 1 and 2 Hz, respectively, with 10 s rest between two frequencies. This was carried out in a perfusion chamber, using the IonOptix Calcium and Contractility System (IonOptix). Subsequently, calcium transients at each frequency were recorded for a period ranging from 5 to 10 s with a 40 × objective. All parameters were calculated offline using the IonWizard 6.3.4 software (IonOptix).

### 2.6 Immunofluorescence staining

Cells were fixed in 4% paraformaldehyde (PFA) for 10 min at room temperature (RT). Subsequently, they were permeabilized with a 0.3% vol/vol Triton X-100 solution for 30 min and then blocked with 3% bovine serum albumin for 1 hour at RT. Afterwards, cells were incubated with cTNT (MA512960, ThermoFisher), α-actinin (A7811, Sigma), γ-H2Ax (05–636-I, Merck) and cleaved caspase (9664, Cell signaling technology) antibody at 4 °C overnight followed with Alexa Fluor 488- or 555-conjugated secondary antibody (A11001 or A31570, ThermoFisher) staining for 1 hour at RT in the dark. Nuclei were stained with DAPI, and thorough washing with PBS was conducted between each treatment step. Images were captured by the Operetta CLS High-Content Analysis System and subjected to analysis using the Harmony 4.9 software.

### 2.7 Fabrication and culture of rat engineering heart tissue (rEHTs)

To generate 1.5 × 8 mm 3D rat cardiac tissue bundles, polydimethylsiloxane (SYLGARD 184) molds were designed and microfabricated, as per previously established methods. For the hydrogel solution, a mixture of 24 µl 10 mg mL^−1^ fibrinogen (F3879, Sigma), 12 µl Matrigel (354277, Corning), and 24 µl 2x medium was prepared. This hydrogel mixture was then combined with 1.5 × 10^6^ neonatal rat cardiomyocytes (NRCMs) in a 58 µl medium. After adding 2.4 µl of 50 U mL^−1^ thrombin (T7201, Sigma), the cell/gel mixture was placed in a mold and kept at 37 °C for 15 min to allow for proper polymerization.

### 2.8 rEHT contractility analysis

To induce rhythmic contractions in the rEHT, a programmable electric stimulator (Chengdu Instrument Factory YC-2) was employed. The stimulator applied an electric field with a voltage of 10 V, resulting in rhythmic stimulation of the rEHT at a frequency of 1.5 Hz. For video analysis of rEHT contractions, recordings were made at a rate of 25 frames per second. Subsequently, the amplitudes of the rEHT contractions captured in the videos were thoroughly analyzed using the MYOCYTER plugin.

### 2.9 RNA-seq library preparation

Total RNA was extracted using the RNazol RT reagent (RN190-200, MRC). Potentially residual DNA was removed by on-column digestion with RNase-free DNase (79254, Qiagen). Subsequently, the generation of transcriptome library was executed using the KAPA Hyper Prep Kit (KK8504, Roche). Sequencing of the amplified libraries was performed on the Novaseq platform (Illumina).

### 2.10 RNA-seq data analysis

Paired-end reads were trimmed with Trimmomatic v.0.36 (Bolger, Lohse et al. 2014) with options: “ILLUMINACLIP:TruSeq3-PE.fa:2:30:10:8:true LEADING:10 TRAILING:10 MINLEN:30″, and only those properly-paired reads after trimming were retained. Clean reads were then mapped against the human reference genome (GRCh38) using HISAT2 (v.2.1.0) software (Kim, Langmead et al. 2015) to generate read alignments for each sample. The quantification of gene expression was performed using featureCounts v.1.6.0 (Liao, Smyth et al. 2014) with parameters “-p -B -T 8 -t exon -g gene_name”. Differential expression analysis was performed with the R package DESeq2 v.1.30.0 (Love, Huber et al. 2014). Gene Ontology analysis was performed using the R package clusterProfiler v.3.18.1 (Yu, Wang et al. 2012). Additionally, Gene Set Enrichment Analysis (GSEA) was performed against MSigDB, GO, Reactome and KEGG with R package clusterProfiler v.3.18.1. Heatmap was generated by the R package pheatmap v.1.0.12 (available at https://CRAN.R-project.org/package=pheatmap).

### 2.11 Animal

All mouse and rat experiments were conducted in accordance with the university guidelines and were approved by the Sun Yat-sen University Animal Care and Use Committee (SYSU-IACUC-2021-B1634) ensuring the highest standards of animal welfare and ethical conduct. Male 1-day-old neonatal Sprague-Dawley rats were purchased from Experimental Animal Center of Sun Yat-sen University. Male C57BL/6 mice were obtained from the Gempharmatech Co.,Ltd (Nanjing, China). Mice within the age range of 8 to 10 weeks were used for the experiments, adhering to established guidelines and protocols.

### 2.12 DOX-IC mouse model and protective drug administration

To induce DOX-IC in C57BL/6 mice, male mice aged between 8 and 10 weeks were used. DOX dissolved in saline was administered intraperitoneally to mice at a dose of 5 mg/kg every week. This treatment regimen was continued for a total duration of 4 weeks, resulting in a cumulative dose of 20 mg/kg. Control mice were injected with the same volume of saline. For experimental groups, Zidovudine (ZIDO) (T1416, TargetMol) at 15 mg/kg or Dexrazoxane (DRZ) (S5651, Selleck) at 50 mg/kg, both dissolved in saline, was administered intraperitoneally to mice 1 hour before DOX injection.

### 2.13 Cardiac function assessment by echocardiography

Mice were anesthetized through inhalation of isoflurane, specifically maintained at a concentration of 1%–1.5%. Transthoracic echocardiography was performed using a Vevo 3100 high-resolution imaging system with 40-MHz (VisualSonics). Echocardiographic M-model images were obtained for the calculation of critical parameters such as left ventricle ejection fraction and fractional shortening, which are indicative of cardiac performance and function.

### 2.14 Histology

Heart samples were collected and fixed in a 4% PFA solution at RT for 1 hour, and then dehydrated through 10%–30% sucrose at RT for 3 h. Subsequently, the heart samples were embedded in the optimal cutting temperature compound (OCT) and sectioned according to the standard cryosection procedures. Haematoxylin and eosin (H&E) and Masson's trichrome (Masson) staining were performed according to standard procedures following the manufacturer's recommendations (G1345-8, Solarbio).

### 2.15 Statistical analysis

Data were presented as mean ± SEM from at least three biologically independent experiments. Statistical tests were performed with Graphpad Prism9. Statistical significance was determined using the one-way analysis of variance (ANOVA) with Turkey's post hoc test for multigroup comparisons. Differences were considered statistically significant with *P* < 0.05.

## 3 Results

### 3.1 High-throughput repurposed drug screening using hCMs reveals small molecules mitigating DOX-induced cardiotoxicity *in vitro*

**Fig. 1:**
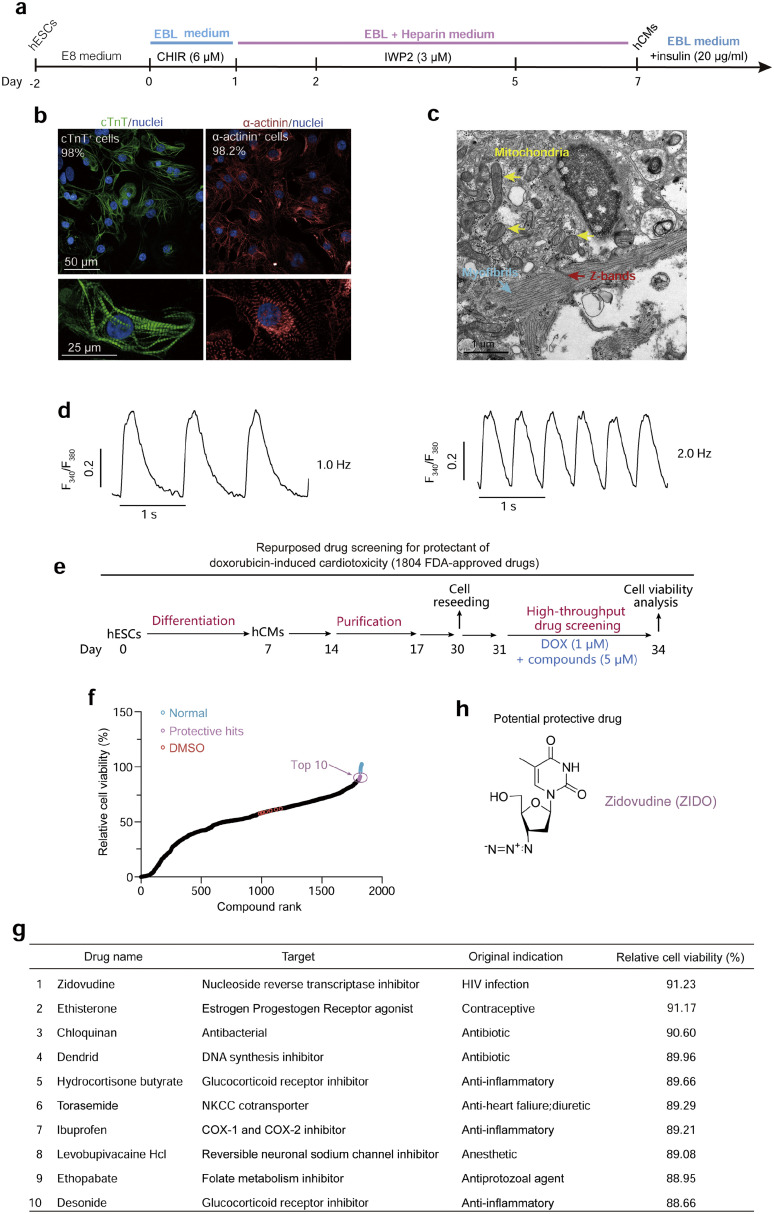
**Discovery of DOX-IC protective drug candidates through hCM-based repurposed drug screening.** a, A chemically defined protocol for cardiac differentiation of human embryonic stem cells (hESCs). hCMs, human CMs; CHIR, CHIR99021; EBL, E8 basal lipid. b, Representative immunofluorescence staining of hCMs culture showing high purity (upper) and abundant sarcomere structures (lower). c, Transmission electron microscopic analyses of hCMs. Myofilaments (blue arrows), Z-bands (red arrows), and mitochondria (yellow arrows) are visible. d, Representative traces of spontaneous intracellular calcium transient of hCMs under electrical field stimulation at 1 Hz and 2 Hz, respectively. e and f, The workflow (e) and primary high-throughput drug screening results (f) of DOX-IC protectants. g, List of top ten screened drug hits based on protection levels. h, Chemical structure of the leading hit Zidovudine (ZIDO).

To efficiently assess cardiotoxicity in a highly enriched population of human CMs, we generated hCMs using a chemically defined system followed by lactate-based metabolic purification as we previously described (Fig. 1a) [25,27,30]. This protocol reproducibly yielded a homogeneous population of hCMs, with purity exceeding 98%, as measured by immunostaining analyses of the CM markers, namely, cardiac troponin (cTNT) and α-actinin (Fig. 1b). These hCMs exhibited the hallmark features of CMs, including well-organized sarcomeres surrounded by numerous mitochondria (Fig. 1b), the characteristic pattern of cardiac calcium transients (Fig. 1c), and rhythmic spontaneous contraction (Supplementary Video 1 online). We reasoned that co-treatment with protective drugs should reduce DOX-induced cell death; hence, we took advantage of the scalable and highly homogeneous hCMs and set up a high-throughput, 384-well format drug screening pipeline based on cell viability as phenotypic readouts (Fig. 1d). Cell viability was assessed by the Calcein-AM/propidium iodide (PI) staining assay and subsequently assessed by a high-content imaging and analytical system. Various plating densities, as well as the concentration and duration of DOX treatment, were optimized for a 384-well format to achieve ∼40% cell death in three days.

In the context of this fine-tuned system, we embarked on the screening of a library comprising 1804 FDA-approved drugs. Each compound was tested at a concentration of 5 µM and was individually administered to the hCMs concomitantly with DOX (Fig. 1e). The primary screen identified ten compounds with cell viability greater than 70%, which were selected for further validation (Fig. 1f). Subsequent examination of these promising hits pinpointed Zidovudine (ZIDO), an antiretroviral drug employed in the treatment of the acquired immune deficiency syndrome (AIDS), to be the most potent and efficacious hit capable of reproducibly safeguarding hCMs against DOX-IC (Fig. 1g). As such, ZIDO emerged as the focal point for the remainder of this study.

### 3.2 ZIDO promotes cell survival and restores calcium handling capacities of hCMs after DOX treatment

**Fig. 2:**
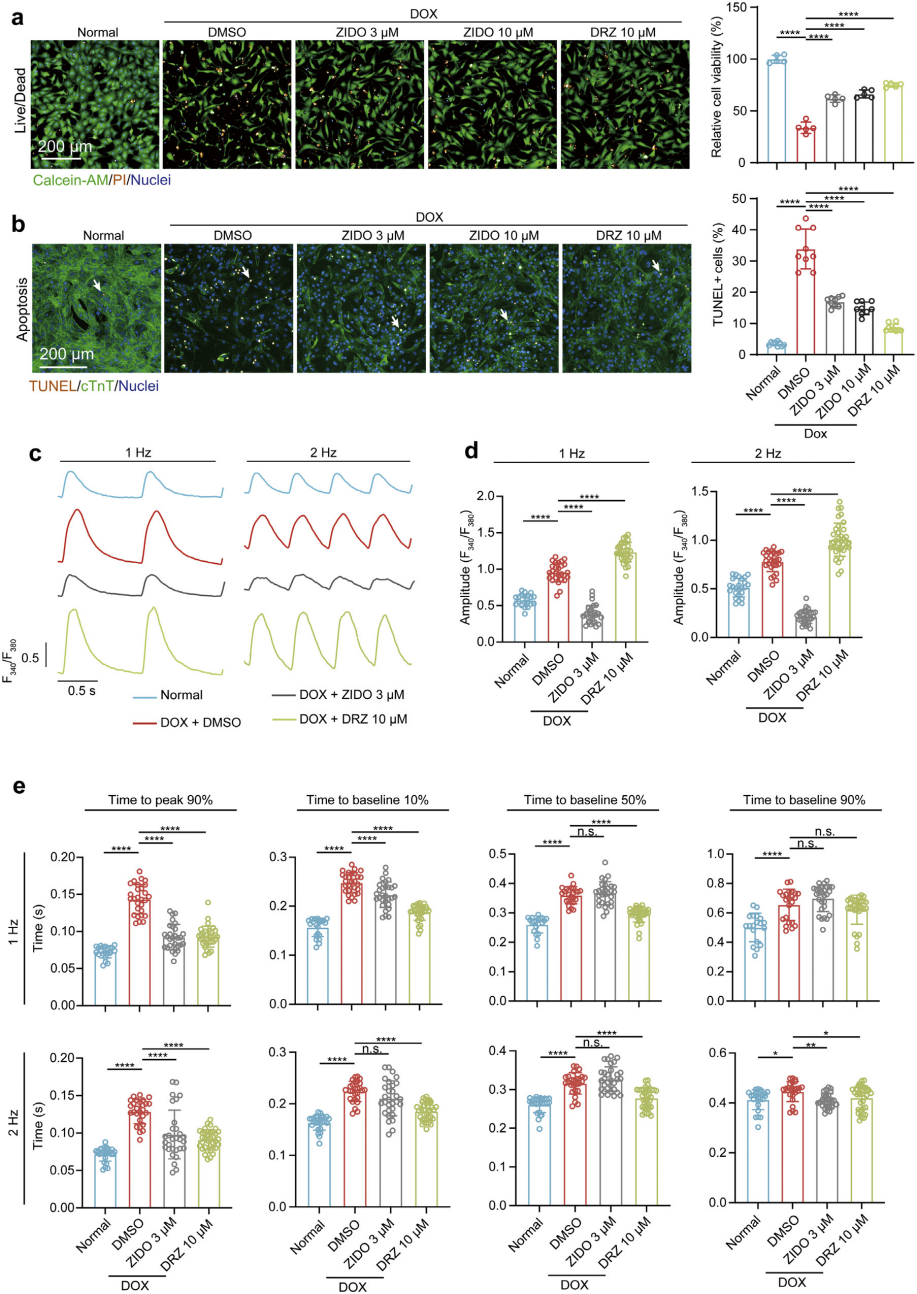
**Assessment of *in vitro* DOX-IC protective effects of ZIDO in hCMs.** a and b, Representative (left) and quantitative (right) analysis of cell viability (by Calcein-AM/PI staining (*n* = 5 experiments; Nine randomly selected images were analyzed for each experiment) and apoptosis by TUNEL assay. (*n* = 9 experiments; 3 randomly selected images were analyzed for each experiment) in DOX-treated hCMs with or without the co-administration of ZIDO or DRZ at the indicated dose. c–e, Representative traces (c) and averaged parameters (d and e) of intracellular calcium transients recorded from hCMs treated with 0.75 µM DOX alone, or DOX plus 3 µM ZIDO or 10 µM DRZ for 48 h, under 1 Hz or 2 Hz electrical field stimulation. *n* = 20–36 cells for each group. Data are presented as mean± SEM. *p < 0.05, **p < 0.01, ***p < 0.001, ****p < 0.0001, n.s., not significant, estimated by one-way ANOVA with Tukey's post hoc test.

ZIDO treatment at clinically relevant concentrations in DOX-injured hCMs offered protective effects comparable to those of dexrazoxane (DRZ), as indicated by a higher number of survived cells and improved cell morphology compared to hCMs stimulated by DOX alone (Fig. 2a). In addition, despite DOX treatment induced almost 30% of the hCMs to undergo apoptosis based on terminal deoxynucleotidyl transferase-mediated dUTP nick end labeling (TUNEL) analyses, ZIDO application notably alleviated apoptosis of hCMs (Fig. 2b and S3). This suggests a meaningful reduction in apoptosis, which is a crucial factor in cardiotoxicity.

Calcium transients are associated with relaxation, contraction, and arrhythmogenicity in CMs. Hence, the dynamic monitoring of calcium alterations is crucial for comprehending CM function and the therapeutic efficacy of drugs [31]. DOX is known to not only induce CM death but also disturbs cardiac function and contraction [22]. Studies have indicated that both DOX and its metabolite, doxorubicinol, can induce calcium release from the sarcoplasmic reticulum, causing calcium overload that leads to contractile disorder [32,33]. To ascertain whether ZIDO could mitigate the adverse effect of DOX on CM function and calcium handling properties, we scrutinized the intracellular calcium transients in hCMs treated with both DOX and ZIDO or with DOX alone for two days. These calcium transients were visualized using the calcium indicator dye Fluo-4, with the drug-treated hCMs subjected to escalating frequencies of electrical field stimulation (1 and 2 Hz) to identify changes in cardiac refractoriness. Consistent with previous reports, DOX treatment triggered robust calcium release from the sarcoplasmic reticulum and induced abnormal large calcium transients characterized by significantly increased amplitudes, under both frequencies of stimulation (Fig. 2c, 2d). Furthermore, DOX treatment notably prolonged the calcium transient durations, evidenced by increased time to peak and time to baseline of the calcium transients (Fig. 2c, 2d). This observation implies that DOX disrupts the calcium handling machinery of hCMs, leading to inefficient uptake and release of calcium stores. It is noteworthy that ZIDO treatment, when administered alone, had no detrimental impact on the calcium handling capacities of hCMs (Fig. S2). Crucially, co-treating the cells with DOX and ZIDO effectively reversed the DOX-induced alterations in calcium transient duration (Fig. 2c, 2d). Additionally, the abnormal increases in calcium transient amplitudes induced by DOX were relieved dramatically by ZIDO treatment, signifying the functional restoration of calcium handling machinery (Fig. 2c, 2d). To further confirm the beneficial effects of ZIDO on myocardial calcium handling and contractile function, we harnessed primary cardiomyocytes from neonatal rats to construct a rat heart engineering tissue (rEHT) model and evaluated the impact of ZIDO on the contractile function of rEHTs that received DOX treatment. In line with our observations in hCMs, ZIDO exerted a significantly positive impact on the contractility of rEHTs following DOX treatment, further validating its effectiveness in improving calcium handling capacity essential for heart contraction (Fig. S1 and supplementary video 2 online). Collectively, these results underscore ZIDO’s capacity to shield human CMs from DOX-IC *in vitro*.

### 3.3 Transcriptional alterations induced by ZIDO treatment in hCMs

**Fig. 3:**
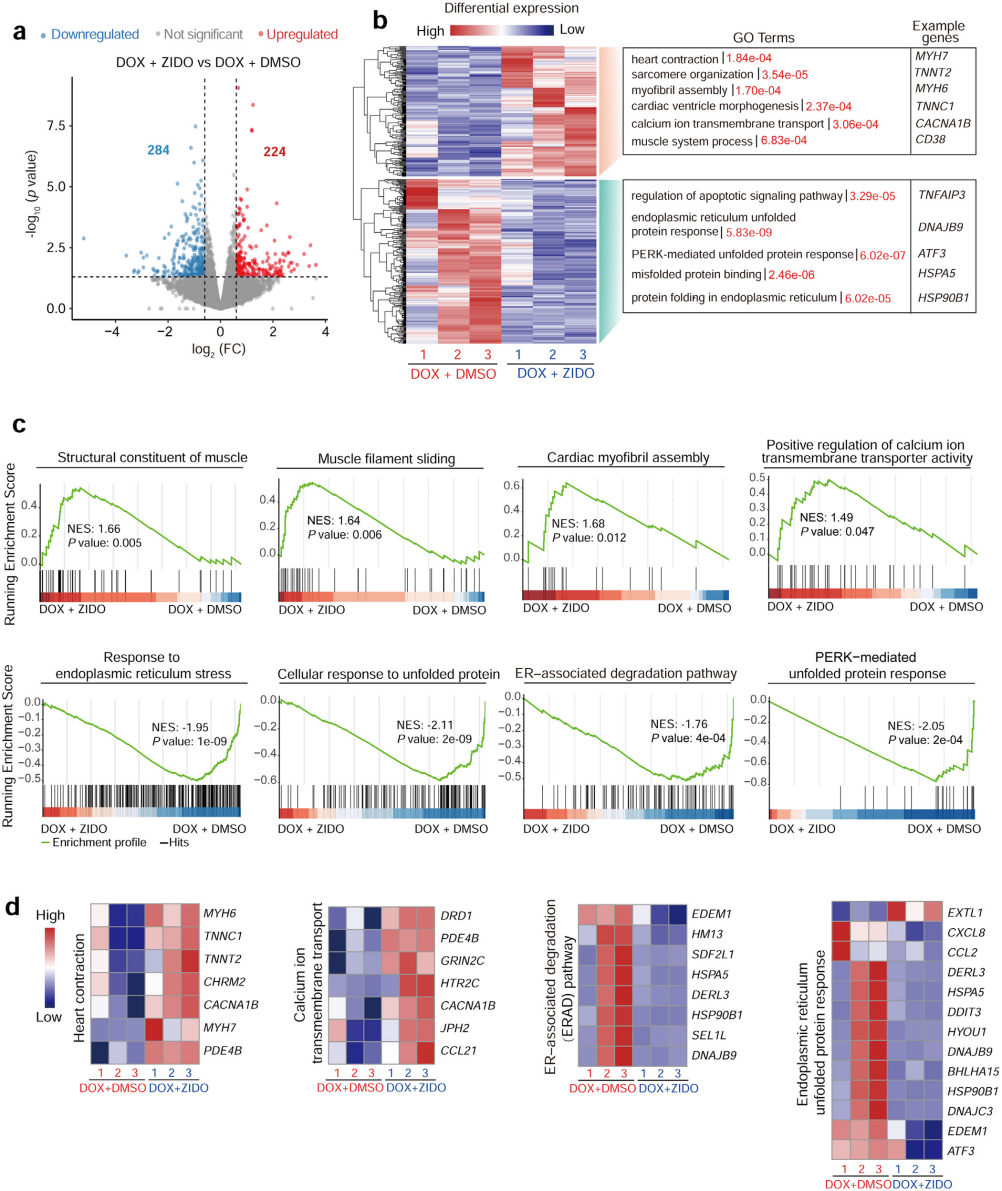
**Transcriptional changes induced by ZIDO.** a, Volcano plot showing the number of differentially expressed genes (*p*-value <0.05 and fold change (FC) >1.5) in DOX-treated hCMs with or without ZIDO administration. b, Two-way hierarchical clustering of all 508 differentially expressed genes identified in (a). Up- and down-regulated genes are clustered and the representative gene ontology (GO) terms enriched in each cluster are listed along with their *p*-values and the example genes. c, Gene set enrichment analysis (GSEA) showing terms enriched in up- or down-regulated genes induced by ZIDO. NES, normalized enrichment score. D, Expression levels of the relevant marker genes revealed by RNA-seq.

To elucidate the underlying biological processes responsible for the protective effects of ZIDO, we conducted RNA sequencing (RNA-seq) analysis on DOX-treated hCMs, both with and without concurrent ZIDO treatment for two days. A pairwise comparison of the transcriptomes of ZIDO and dimethyl sulfoxide (DMSO)-treated cells unveiled numerous differentially expressed genes, with 224 genes showing significant upregulation and 284 genes displaying downregulation (Fig. 3a). Gene ontology (GO) enrichment analysis demonstrated that the upregulated genes induced by ZIDO were mainly correlated with heart contraction. These included genes important for sarcomere organization and ion homeostasis (Fig. 3b). In contrast, ZIDO treatment downregulated genes linked to cell death and cellular stress, including key regulators of apoptotic and unfolded protein response (UPR) signaling pathways (Fig. 3b), both of which are hallmark features of DOX-IC [34,35]. These results were independently corroborated by the Gene Set Enrichment Analysis (GSEA), in which a strong enrichment of cardiac contraction genes was observed in the ZIDO-treated cells, while simultaneously reducing the expression of UPR-related genes that were notably elevated in the control hCMs (Fig. 3c). Subsequently, we evaluated a panel of genes involved in heart contraction, ion homeostasis, and endoplasmic stress response. Our analysis revealed that ZIDO did affect these genes integral to these cellular functions (Fig. 3d). Excessive or persistent endoplasmic reticulum (ER) stress can result in the buildup of unfolded or misfolded proteins, consequently triggering the activation of endoplasmic reticulum sensory molecules, which, in turn activate the corresponding UPR signaling pathways. The UPR initiates and mediates apoptosis, and can contribute to the onset and progression of various diseases. Numerous studies have implicated ER stress in the development of a range of cardiovascular diseases (CVDs), positioning it as an important therapeutic target for CVDs [[36], [37], [38]]. We therefore speculate that ZIDO may protect hCMs from DOX-induced injury by mitigating UPR-mediated cell death. To test this hypothesis, we co-treated hCMs with different concentrations of inhibitors targeting RNA-dependent protein kinase-like ER kinase (PERK, using GSK2606414) or inositol-requiring protein α (IRE1,with 4µ8c), both in combination with DOX. PERK and IRE1 are key sensors in the cell death signaling pathway regulated by UPR. Our findings revealed that the inhibitors of PERK and IRE1 could recapitulate the protective effects of ZIDO and significantly enhanced cell viability with comparable protective effects to ZIDO (Fig. S4). These results at least partly suggest that ZIDO may safeguard hCMs from DOX-induced injury by alleviating UPR-mediated cell death. Taken together, ZIDO may employ multiple mechanisms to combat DOX-IC via preserving the contractile function and alleviating UPR-mediated cellular stress and death.

### 3.4 ZIDO alleviates DOX-IC in a mouse model of DOX-induced chronic heart injury

**Fig. 4:**
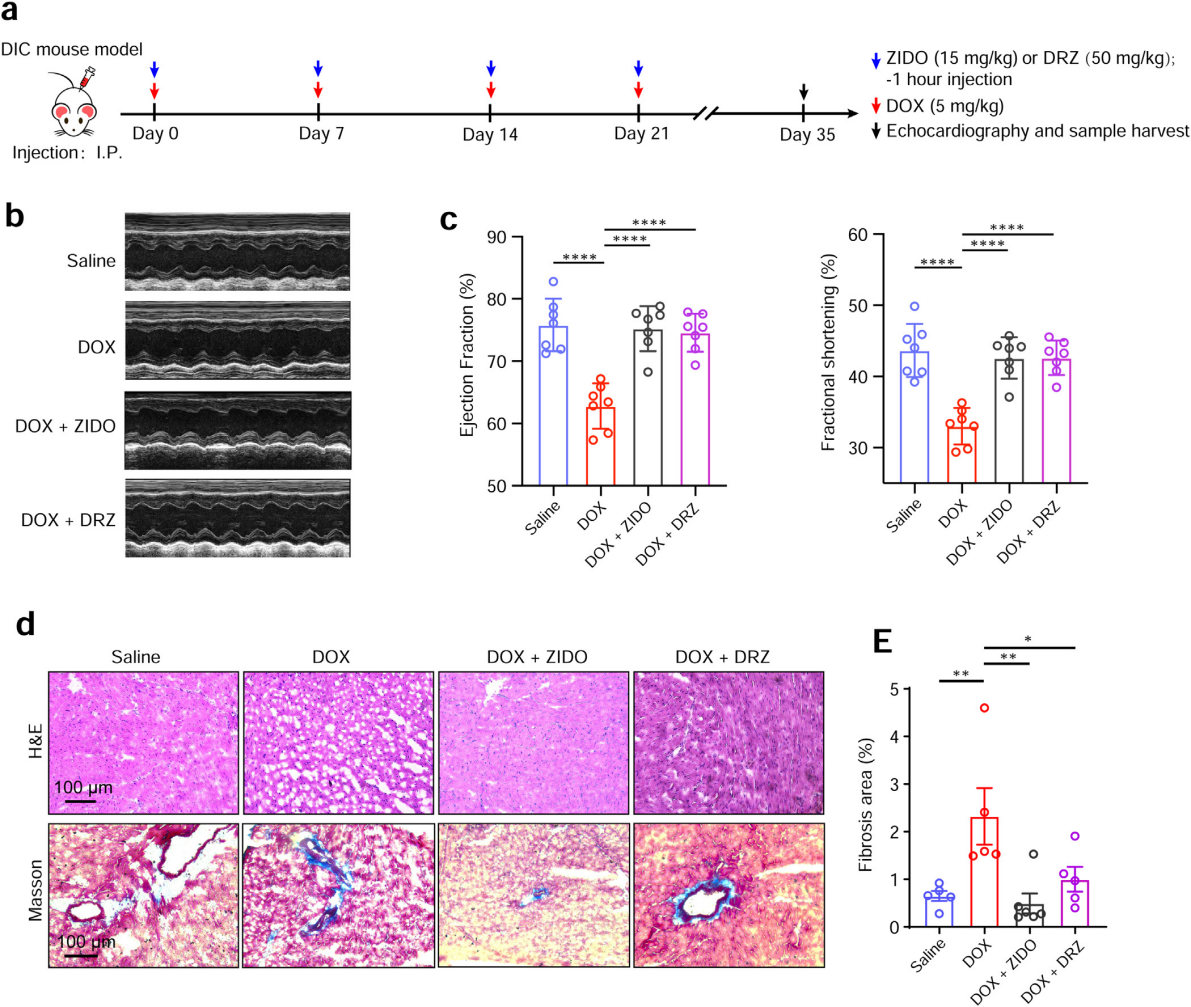
**Assessment of the therapeutic efficacy of ZIDO in a mouse model of DOX-induced chronic heart injury.** a, Schematics showing time points when mice received peritoneal injections, subsequently analyzed with echocardiography, and finally sacrificed for heart sample collections. b and c, Representative M-mode echocardiography images (b) and statistical analysis (c) of the ejection fraction and fractional shortening of mouse hearts (*n* = 7 mice for each group). d, Hematoxylin and eosin (H&E) and Masson staining of heart section samples. e, Statistical analysis of the fibrotic area in Masson-stained heart section samples (*n* = 5 or 6 mice for each group). Data are presented as mean ± SEM. *p < 0.05, **p < 0.01, ***p < 0.001, ****p < 0.0001, n.s., not significant, estimated by one-way ANOVA with Tukey's post hoc test.

In humans, chronic accumulation of DOX can lead to heart failure. To further investigate whether ZIDO exhibits a cardiac protective effect against DOX-IC *in vivo*, we next assessed its therapeutic efficacy in a mouse model of DOX-induced chronic heart injury. Adult mice received a cumulative dose of DOX at 20 mg/kg over four intraperitoneal injections with weekly interval and were analyzed after one month (Fig. 4a). ZIDO (15 mg/kg) or saline as a control were intraperitoneally injected into the mice one hour before each time of DOX injection. DRZ (50 mg/kg) was similarly administrated as ZIDO and served as a positive control. High-resolution echocardiographic analysis revealed a significant reduction in heart function, indicated by decreased left ventricular ejection fraction and fractional shortening following DOX treatment. Notably, ZIDO exhibited a protective effect comparable to that of DRZ, almost completely reversing the DOX-induced decline of cardiac function at four weeks (Fig. 4b, 4c).In line with these findings, histological analysis demonstrated loose myocardial structure and elevated level of fibrosis in the DOX-treated hearts. In contrast, ZIDO treatment reduced the extent of fibrosis compared to the saline control and restored normal tissue organization (Fig. 4d). Taken together, these data demonstrate that ZIDO prevents DOX-IC *in vivo* and serves as a novel therapeutic reagent for the treatment of DOX-induced chronic heart injury.

## 4 Discussion

In this study, we employed a comprehensive high-throughput phenotypic screening approach involving a repertoire of FDA-approved drugs. This strategy led to the identification and subsequent validation of ZIDO as a promising novel candidate for mitigating DOX-IC in both *in vitro* and *in vivo* settings. Our in-depth RNA-seq analyses suggest that ZIDO may protect the CMs against DOX-IC by down-regulating the UPR-mediated cell death induced by DOX treatment.

Following the identification of ZIDO as a promising candidate for mitigating DOX-IC, our focus shifts to the intricate landscape of drug development, a terrain fraught with formidable challenges. A significant obstacle, often insurmountable, lies in the realm of cardiotoxicity. Indeed, it is disheartening to note that a mere quarter of cardiovascular drugs successfully pass phase 1 clinical trials [39]. Moreover, considering the average investment on a clinically applicable drug takes nearly 2.5 billion dollars and an average 10–12 years [28], the urgency to bolster the drug discovery success rate becomes evident. In this regard, the avenue of drug repurposing emerges as an enticing alternative, characterized by a reduced probability of failure, expedited development, and diminished financial burdens [29,40].

Successful repurposing is contingent on appropriate models. Traditionally, drug discoveries have been hindered by interspecies disparities between human and animal models. It is estimated that the translational rate from animal experiments to human clinical trials is lower than 8% [41]. This is exemplified by a recent report showing that statin fails to prevent heart function decline in clinical trials [42], despite statin being previously reported to offer DOX-IC attenuation in a relevant mouse model [43]. Directed differentiation from hPSCs can in theory generate any cell type in the body, rendering it a potent tool for identifying drug candidates with therapeutic benefits that may have been previously overlooked. This enables us to successfully harness drug repurposing approaches on various systems [27,44]. A recent breakthrough in the discovery of prophylactic drugs against SARS-CoV-2 [45] serves as another encouraging example of drug repurposing through the utilization of a humanized system.

ZIDO, an anti-retrovirus drug first discovered for treating HIV and avian influenza virus (AIVs) in the 1980s [46], holds the distinction of being included in the World Health Organization's List of Essential Medicines [47]. As a nucleoside analog, it works by inhibiting reverse transcription, thereby terminating viral DNA-chain elongation [48]. In addition, at the time when ZIDO was first synthesized in the 1960s, it was designed for cancer treatment, as cancer cells usually express telomerase with reverse transcriptase activities. Furthermore, 3′-azido-3′-deoxythymidine-5′-triphosphate, an active metabolite of ZIDO, has been shown to inhibit HIV-reverse transcriptase in kinetic analyses, prioritizing it over cellular DNA polymerases [[49], [50], [51]]. This evidence collectively points toward the compound's enhanced selectivity for viruses over normal human cells, therefore sparking inquiry into how it exerts its effects on hCMs. Our RNA-seq analysis in this study offers insights into a potential mechanism, suggesting that ZIDO may confer protection to CMs by dampening global protein translation. This is of significance because DOX is known to trigger the production of misfolded proteins [35], although a more detailed mechanism remains unknown and needs to be investigated further. It is worth noting that while ZIDO demonstrates protective effects when co-administered with DOX, whether it could reverse DOX-IC when administered after the onset of the disease remains unassessed, and further studies are warranted.

In summary, our drug repurposing approach has led to the discovery of ZIDO as a promising protective compound against DOX-IC from clinically available drugs. ZIDO has demonstrated effectiveness both *in vitro* and *in vivo*. This strategy represents an example of discovering repurposed drugs for severe diseases currently without appropriate remedies and may accelerate their translation to clinical studies in the near future.

## Declaration of competing interest

The authors declare that they have no conflicts of interest in this work.

## Appendix Supplementary materials

Supplementary Video 1 Rhythmic spontaneous contraction of hCM. Image, application 1

Supplementary Video 2 Rhythmic contraction of rEHTs stimulated under electric field. Image, video 2 dummy alt text

Image, application 3
