# Supplementary material for: Discovery of Zidovudine as a cardiomyocyte protectant for doxorubicin-induced toxicity through high-throughput phenotypic drug screening
Source: Fundam Res. 2023 Nov 19;6(2):885–94. doi: 10.1016/j.fmre.2023.10.010 (PMC13069625; doi:10.1016/j.fmre.2023.10.010)
Supplement: Supplementary file 1 — Supplementary Video 1 Rhythmic spontaneous contraction of hCM. [file mmc1.pdf]

Figure S1.

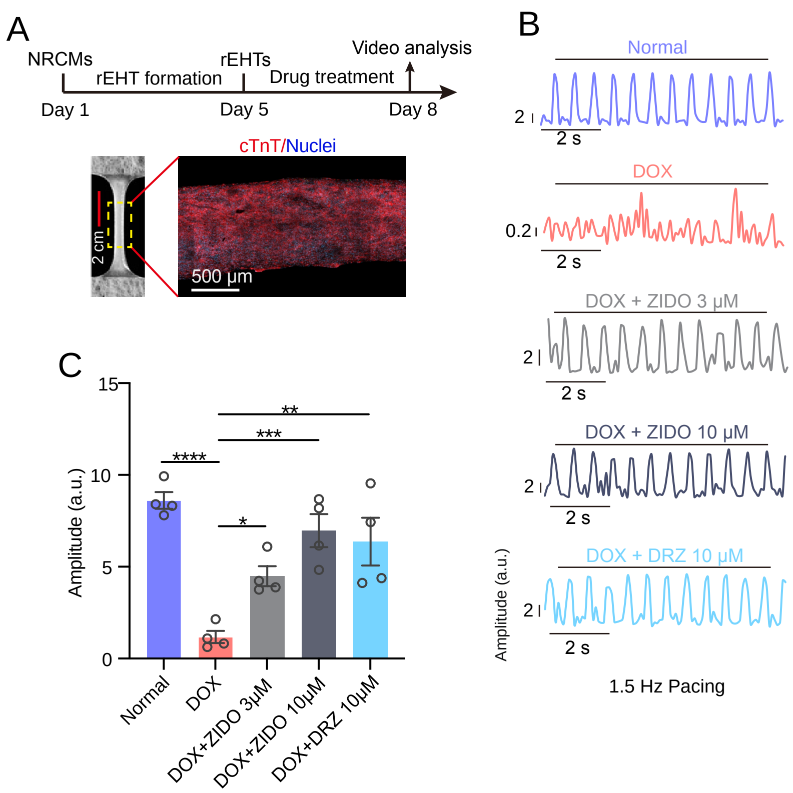

Figure S1. Assessment of DOX-IC protective effects of ZIDO in rEHTs.

(A) Schematics of the rEHT-based assays and the representative rEHT morphology.B-C, Representative traces (B) and averaged parameter (C) of contractility recorded from NRCMs treated with 0.1 μM DOX along, DOX plus 3 μM ZIDO, or 10 μM DRZ for 72 hours, under 1.5 Hz electrical field stimulation. n=4 rEHTs each group. Data are presented as mean± SEM. *$p < 0.05$, **$p < 0.01$, ***$p < 0.001$, ****$p < 0.0001$, n.s., not significant, estimated by one-way ANOVA with Tukey's post hoc test.

Figure S2.

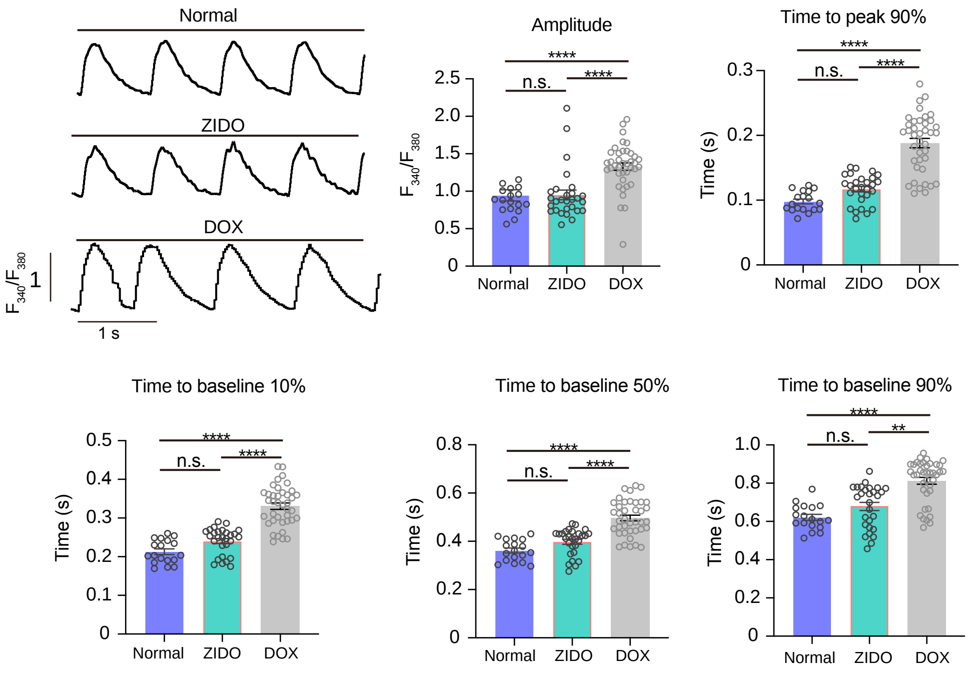

Figure S2. ZIDO has little effects on hCMs function under electrical field stimulation.

Representative traces and averaged parameters of intracellular calcium transient in hCMs treated with 10 μM ZIDO or 0.75 μM DOX for 48 hours. n=19-39 cells for each group. Data are means ± SEM. *$p < 0.05$, **$p < 0.01$, ***$p < 0.001$, ****$p < 0.0001$, n.s., not significant, estimated by one-way ANOVA with Tukey's post hoc test.

Figure S3.

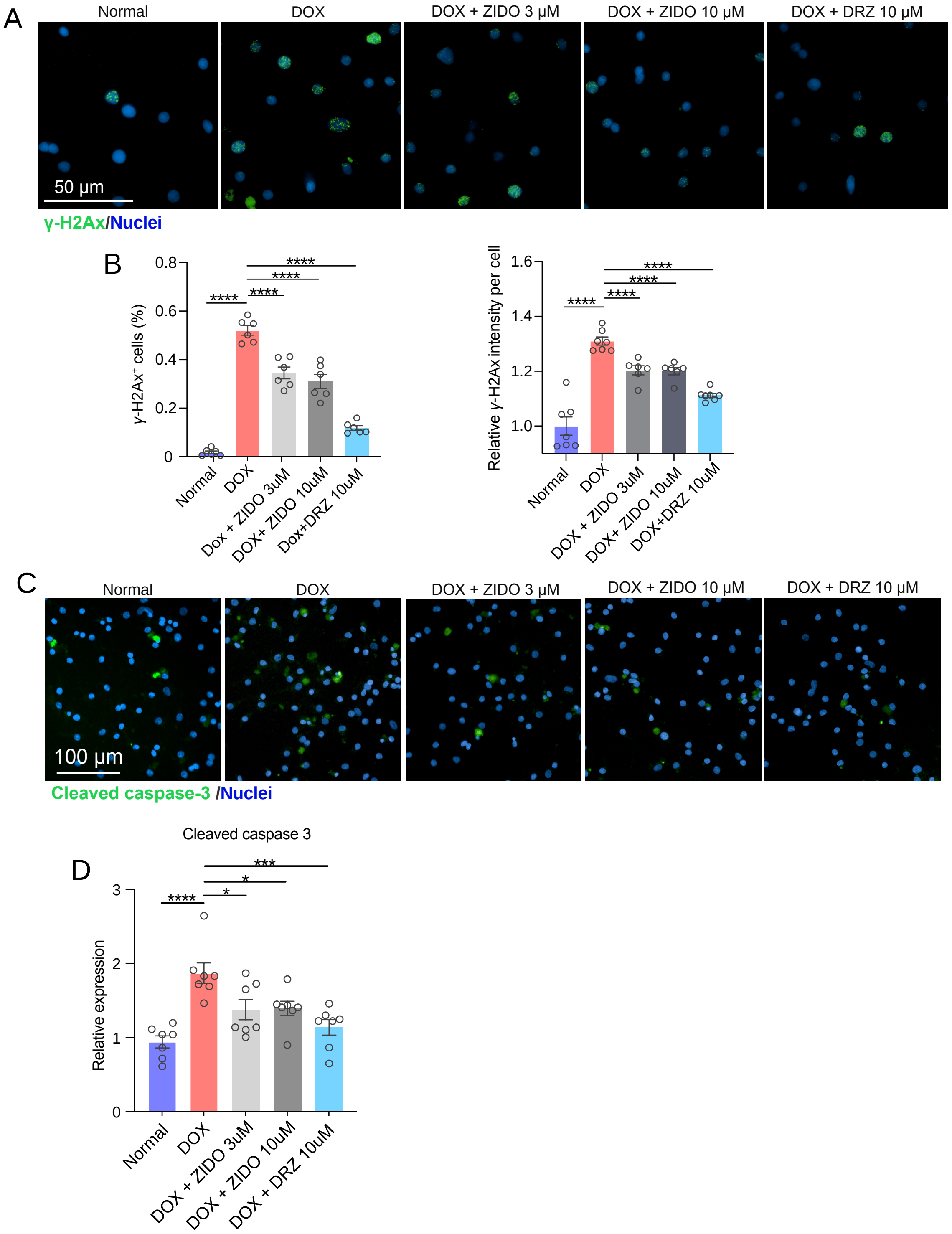

Figure S3. ZIDO alleviates DNA damage and apoptosis mediated by cleaved caspase-3 in hCMs related to figure 2.
(A-D) Representative and quantitative analysis of DNA damage repair (by γ-H2Ax staining. n=7 replicates.10 images were analyzed for each replicate), apoptosis (by caspase 3 cleaved staining. n=7 replicates.10 images were analyzed for each replicate), in hCMs treated with 0.75 μM DOX alone, DOX plus 3 μM ZIDO, or 10 μM DRZ for 24 hours (A-B) or 48 hours (C-D). Data are means ± SEM. *$p < 0.05$, **$p < 0.01$, ***$p < 0.001$, ****$p < 0.0001$, n.s., not significant, estimated by one-way ANOVA with Tukey's post hoc test.

Figure S4.

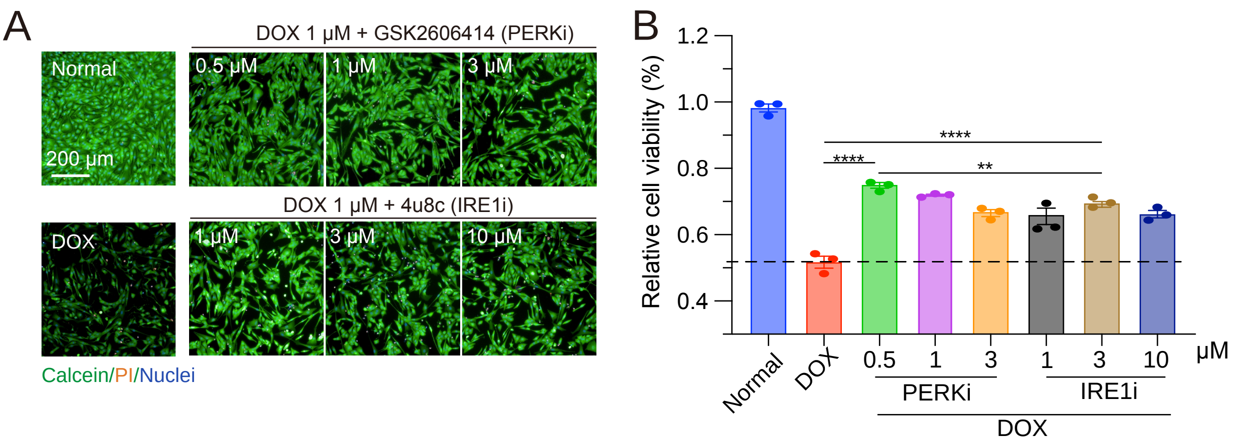

Figure S4. Inhibition of PERK and IRE1 signaling phenocopy ZIDO to improve cell viability under DOX treatment.
(A-B) Representative and quantitative analysis of cell viability (by Calcein-AM/PI staining. n=3 replicates.) in hCMs treated with 1 μM DOX alone, or DOX plus 0.5 μM, 1 μM, or 3 μM PERK inhibitor (PERKi, GSK2606414), or 1 μM, 3 μM, or 10 μM IRE1 inhibitor (IRE1i, 4μ8c) for 3 days. Data are means ± SEM. *$p < 0.05$, **$p < 0.01$, ***$p < 0.001$, ****$p < 0.0001$, n.s., not significant, estimated by one-way ANOVA with Tukey’s post hoc test.
